# Efficacy analysis of large-channel spinal endoscope unilateral laminotomy decompression for the treatment of multilevel cervical spinal stenosis with ligamentum flavum hypertrophy

**DOI:** 10.3389/fsurg.2025.1728927

**Published:** 2025-12-08

**Authors:** Yiping Zheng, Luyang Wang, Mingwang Zhao, Yongchun Zhang, Donglin Yang, Xiaoxin Chen, Xingchen Li, Yusheng Xu

**Affiliations:** 1 Department of Orthopaedics, The First Affiliated Hospital of Zhengzhou University, Zhengzhou, China; 2 The First Clinical Medical College of Zhengzhou University, Zhengzhou, China

**Keywords:** multilevel cervical spinal stenosis, large-channel endoscope, open-door laminoplasty, ligamentum flavum hypertrophy, efficacy

## Abstract

**Objective:**

Cervical spinal stenosis predominantly affects the elderly. After 40 years of age, aging induces progressive loss and rupture of elastic fibers in the ligamentum flavum, accompanied by abnormal proliferation and cross-linking of collagen fibers, as well as calcium salt deposition and even ossification. This study investigates the clinical efficacy and safety of large-channel endoscopic unilateral laminotomy decompression for the treatment of multilevel cervical spinal canal stenosis.

**Methods:**

A retrospective study was conducted on 36 Cervical spinal canal stenosis patients with radiologically confirmed who underwent surgical treatment between January 2020 and December 2023.Patients were divided into two groups according to the surgical method: endoscopic group (*n* = 16) and open group (*n* = 20).Perioperative Parameters (operative duration, incision length, intraoperative blood loss, hospitalization period were record and Clinical efficacy were systematically assessed using validated metrics: Visual Analog Scale, Japanese Orthopaedic Association score, Neck Disability Index. Radiographical parameters [C2–C7 Cobb angle, T1 slope, pavlov ratio (canal/vertebral body diameter)] are used to assess the decompression effect and stability of cervical spine.

**Results:**

The endoscopic group demonstrated significant advantages over the open group in operative time (1.6 ± 0.6 vs. 2.1 ± 0.2 h, *P* < 0.05), incision length (1.3 ± 0.1 vs. 9.5 ± 0.7 cm, *P* < 0.05), blood loss (12.4 ± 7.4 vs. 64.3 ± 19.5 mL, *P* < 0.05), and hospitalization duration (6.6 ± 1.1 vs. 8.6 ± 1.4 days, *P* < 0.05). Both groups showed significant postoperative improvements in VAS, JOA, and NDI scores compared to preoperative baselines (*P* < 0.05). At 1 month postoperatively, the endoscopic group exhibited superior VAS scores to the open group (2.69 ± 0.79 vs. 4.4 ± 0.88, *P* < 0.05), though no significant differences were observed at other time points. Radiographic outcomes at final follow-up revealed significantly better cervical Cobb angle (13.57 ± 2.29° vs. 16.34 ± 2.95°, *P* < 0.05) and T1 slope (22.62 ± 1.51° vs. 25.24 ± 2.41°, *P* < 0.05) in the endoscopic group. Conversely, the open group demonstrated greater postoperative spinal canal area and Pavlov ratio (*P* < 0.05). Complications included 2 cases of C5 nerve root palsy and 1 case of axial pain in the open group, while the endoscopic group had 1 case of transient muscle weakness. No reoperations were required.

**Conclusion:**

The large-channel endoscopic unilateral laminotomy decompression demonstrates satisfactory short-term efficacy in treating multilevel Cervical Spinal Stenosis with ligamentum flavum hypertrophy. This minimally invasive technique offers significant advantages including reduced surgical trauma, accelerated recovery, enhanced postoperative cervical stability and relatively higher patient satisfaction.

## 1 Introduction

Cervical spondylosis is a common disease characterized by degenerative changes in the cervical spine affecting the neurovascular structures, which is classified into vertebral artery, radiculopathy, myelopathy, and other subtypes (1). Cervical spinal canal stenosis caused by ligamentum flavum hypertrophy has a high risk of disability due to its dynamic compression mechanism. The most common level is C5–6, followed by C6–7, C4–5, C3–4, and C2–3 (2, 3). With social progress and changes in lifestyle, the incidence of cervical spinal stenosis has shown a significant upward trend. Multilevel cervical spinal stenosis accounts for 8%–10% of all cases of cervical spondylosis and is becoming more prevalent among younger individuals (4). This condition severely impacts patients' quality of life while presenting significant challenges to public health system (5).

In the past, the treatment of cervical spinal stenosis—particularly ligamentum flavum hypertrophy—mainly relied on open posterior cervical surgery including open-door laminoplasty, double door laminoplasty and cervical laminectomy (6–8). Among these, open-door laminoplasty has gradually been adopted by an increasing number of scholars worldwide due to its low surgical difficulty and good results. However, this operation could cause significant damage to the muscles and ligaments in the posterior cervical region during surgery and many patients experience axial pain and other symptoms after surgery (9).

With the development of minimally invasive surgery, the application of spinal endoscopy in the cervical spine has become increasingly widespread. A typical example is the “keyhole” surgery for the treatment of cervical radiculopathy (10). In the case of cervical single-level ligamentum flavum hypertrophy, the spinal endoscopy offers significant advantages in terms of more precise localization and minimally invasive procedures. Additionally, the endoscopy group showed significant improvement in JOA scores after surgery and shorter hospital stays (11). The incidence of axial symptoms after surgery was also significantly lower in the endoscopic group than in the open-door laminoplasty group. In addition, the large-channel spinal endoscope offers unique advantages. First, the 10 mm working channel provides a wider visualization compared to the 6.3 mm working channel, reducing blind spots during surgery and lowering the potential risk of breaking through the ligamentum flavum and entering the spinal canal which could damage the spinal cord. Secondly, endoscopic surgery can achieve bilateral decompression through a unilateral incision by adjusting the angle of the working channel, which is particularly suitable for patients with bilateral spinal canal stenosis (12–14). However, applying this technique to multilevel spinal canal stenosis and its impact on postoperative cervical stability require further investigation.

We conducted a retrospective cohort study targeting patients with multilevel spinal canal stenosis primarily characterized by ligamentum flavum hypertrophy. We analyzed the clinical and imaging data of patients who underwent large-channel spinal endoscope unilateral laminotomy decompression at our hospital from January 2020 to December 2023, and compared them with patients who underwent open-door laminoplasty during the same period. The aim of this study is to preliminarily assess the safety and efficacy of this surgical technique, providing a reference basis for technical improvements and widespread adoption.

## 2 Materials and methods

This retrospective study was approved by the Ethics Committee of the First Affiliated Hospital of Zhengzhou University (Approval No. 2023-KY-1201) and was conducted in accordance with the principles outlined in the Declaration of Helsinki.

### 2.1 Patient inclusion and exclusion criteria

(1) Diagnosis of multilevel cervical spinal canal stenosis (involving ≥2 levels) and inadequate response to conservative treatment; (2) radiographic evidence reveal cervical spinal canal stenosis characterized by “clamp-type” compression or primarily due to ligamentum flavum hypertrophy (ligament thickness ≥3 mm on T2-weighted MRI with compressing the dural sac; (3) Age ≥18 years; (4) provision of written informed consent form; (5) complete follow-up data is available.

### 2.2 Exclusion criteria

(1) Spinal canal stenosis caused by other etiologies (e.g., disc herniation occupying >50% of the spinal canal cross-sectional area, traumatic injury, or neoplastic compression); (2) patients with a history of cervical spine surgery; (3) patients with cervical spine tumors, infections, or other conditions causing symptoms similar to cervical spondylosis; (4) pregnancy or active lactation; (5) patients who are unable or unwilling to comply with the study requirements.

### 2.3 Baseline information

We collected data on 36 patients with multilevel cervical spinal canal stenosis who underwent surgical treatment in our hospital between January 2020 and December 2023.Patients were divided into two groups according to the surgical method: endoscopic group (*n* = 16) and open group (*n* = 20). All patients were strictly included according to the criteria.

### 2.4 Surgical procedure

#### 2.4.1 Endoscopic group

**Figure 1:**
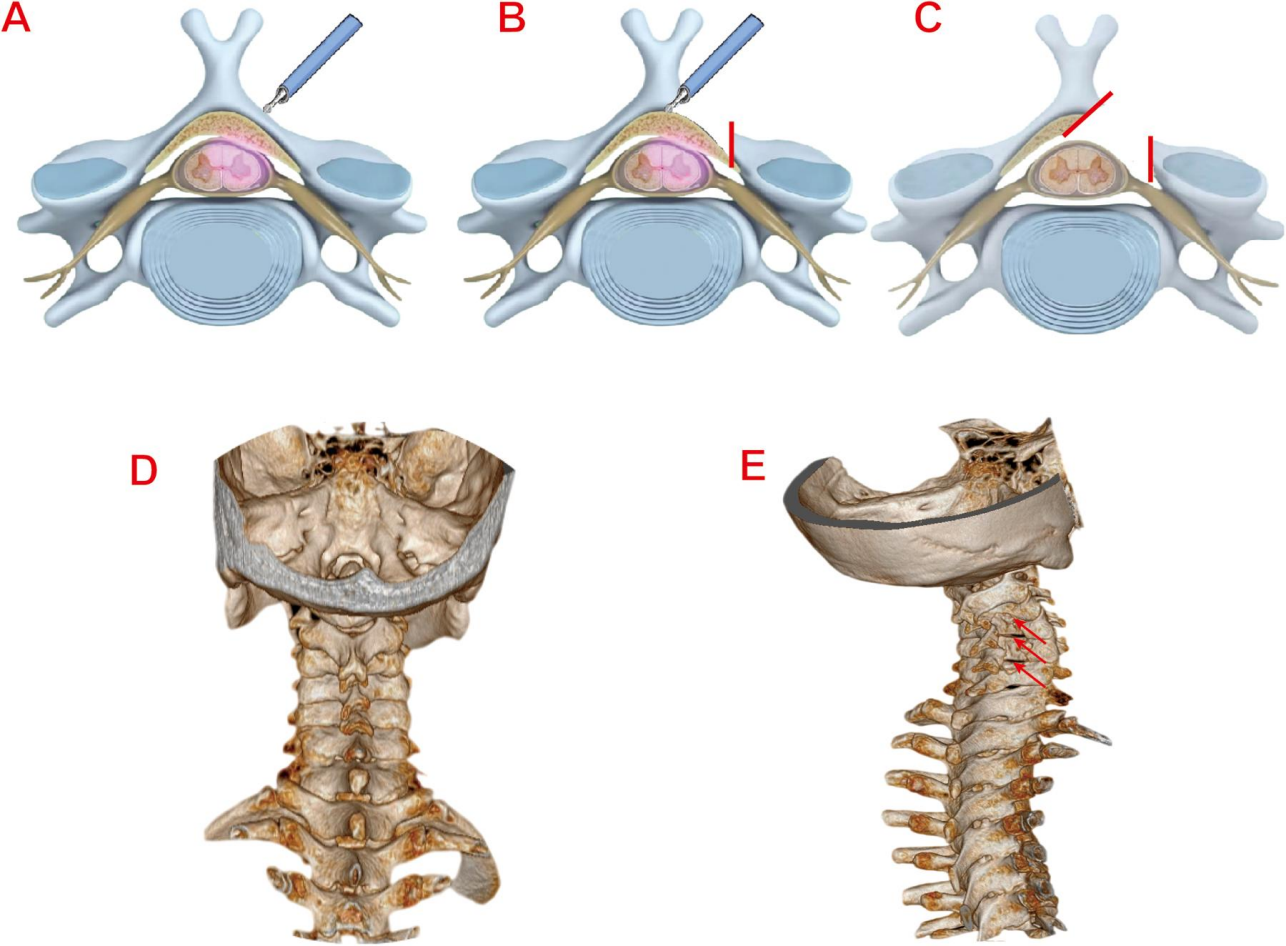
**(A)** Localization of the responsible segment of the vertebral lamina. **(B)** Grinding and polishing of the vertebral lamina with diamond abrasor. **(C)** Complete relaxation of the dura and nerve roots post-decompression. **(D)** Preoperative 3D CT reconstruction image of the cervical spine (Typical Case 1). **(E)** Postoperative 3D CT reconstruction image of the cervical spine (Typical Case 1).

Under general anaesthesia, patients were positioned prone with cervical flexion. After head/shoulder fixation and sterile draping, the target levels were localized using C-arm fluoroscopy. Make a 1 cm skin incision (0.5–1 cm lateral to the midline of the spinous process).The dilation catheter and working tube were inserted in sequence to reach the surface of the lamina with blunt soft-tissue dissection. Under fluoroscopic guidance, the position of the working tube was confirmed to be accurate before connecting to the endoscopic system. The soft tissues on the surface of the vertebral lamina and the root of the spinous process were cleaned and the bleeding was stopped by radiofrequency electrocoagulation, which exposed the root of the spinous process, the same-side lamina. Using diamond abrasor to polish lamina to a transparent thin layer- initially addressing the rostral margin of the inferior vertebral lamina in an inside-out trajectory, followed by the caudal margin of the superior vertebral lamina. After fully exposing the ligamentum flavum, remove it with nucleus pulposus forceps and use laminectomy rongeur to fully decompress. The same method is used for handling other areas of responsibility. In cases of bilateral cervical spinal canal stenosis, the contralateral ligamentum flavum could be resected via the “over-the-top” technique. After adequate decompression, the spinal cord and nerve roots are completely relaxed and pulsation is good. The endoscope system is then withdrawn. Skin incisions are closed in layers after standard antisepsis. The surgical illustration is demonstrated in Figure 1.

#### 2.4.2 Open group

After general anaesthesia induction, patients were positioned prone with head and shoulders immobilized. Following sterile preparation and draping, a 10 cm midline incision was made from C3 to C7 level. Skin, subcutaneous tissue, and deep fascia were incised sequentially. Paraspinal muscles were bluntly dissected bilaterally to expose spinous processes and laminae. After confirming vertebral levels: C3–C7 spinous process bases were resected. The side with more severe symptoms is the open-side, and the other side is the hinged-side. Hinged-side laminae were thinned to unicortical bone using a rongeur. Open-side laminae underwent full-thickness resection. Drive anchors with wires into the hinged side, then lift the vertebral lamina on the side of the open-side and secure it with wire. After fully expanding the spinal canal and thoroughly decompressing it, pulsation of the dura mater was observed, with no obvious compressive objects. The incision was irrigated, bleeding was thoroughly stopped, gelatin sponges were placed, and pre-trimmed autologous bone was inserted on the hinge side. After placing the drainage tube, the incision was closed layer by layer.

### 2.5 Efficacy evaluation

Demographic and operative parameters were recorded for both groups: including Age, sex, body mass index (BMI), Comorbidities (e.g., hypertension), Number of involved vertebral levels, Incision length, operative time, intraoperative blood loss, hospital stay. Clinical outcomes were assessed preoperatively and at 1, 3, 6, 12 months postoperatively using: Visual Analog Scale (VAS) for neck/upper extremity pain, Neck Disability Index (NDI), Japanese Orthopaedic Association (JOA) score. Neurological recovery rate was calculated at final follow-up: $\mathrm{Improvement}\;\mathrm{Rate}=\frac{(\mathrm{Postop}\;\mathrm{JOA}-\mathrm{Preop}\;\mathrm{JOA})}{(17-\mathrm{Preop}\;\;\mathrm{JOA})}$. All radiographic parameters were independently measured by two orthopaedic surgeons to ensure reproducibility of the results.

### 2.6 Radiographic parameter measurement

All cervical x-ray and CT scans were taken strictly following standardized protocols to ensure image accuracy. The measurement parameters are as follows: the C2–C7 Cobb angle is measured to assess the overall physiological curvature of the cervical spine, from the C2 to the C7 cervical vertebrae. The T1 Slope measures the curvature of the first thoracic vertebra to assess angular changes at the cervical-thoracic junction and evaluate sagittal plane balance in the cervical spine. The Pavlov ratio assesses the relative degree of cervical spinal stenosis by measuring the ratio of the anterior-posterior diameter of the spinal canal to the anterior-posterior diameter of the vertebral body at the cervical segment level. All radiographic parameters are independently measured by two orthopedic physicians to ensure reproducibility.

### 2.7 Statistical analysis

Statistical analyses were performed using SPSS Statistics (version 26.0; IBM Corp.) Continuous data are presented as mean ± standard deviation, while categorical data are expressed as frequencies and percentages. Normality of continuous variables was assessed using the Shapiro–Wilk test. Intergroup comparisons of normally distributed data were conducted with independent samples *t*-tests, and multigroup analyses used one-way ANOVA. Non-normally distributed quantitative data were analyzed with the Wilcoxon rank-sum test and reported as median. Categorical data were evaluated using Pearson's *χ*^2^ or Fisher's exact tests for nominal variables and Mann–Whitney *U* tests for ordinal variables. *P* < 0.05 was defined a statistically significant difference.

## 3 Results

### 3.1 Basic parameters and perioperative information

A total of 36 patients were enrolled, including 16 in the endoscopic group and 20 in the open group. Basic parameters and perioperative information of the two groups are summarized in Table 1. No significant differences were observed in demographic or clinical baseline parameters between the two groups (*P* > 0.05). All procedures were successfully completed without intraoperative complications and post-operative infections. All incisions achieved good healing. The endoscopic group had significantly shorter surgical times (1.6 ± 0.6 h vs. 2.1 ± 0.2 h), smaller incision lengths (1.3 ± 0.1 cm vs. 9.5 ± 0.7 cm), less blood loss (12.4 ± 7.4 mL vs. 64.3 ± 19.5 mL), and shorter hospital stays (6.6 ± 1.1 days vs. 8.6 ± 1.4 days) than the open surgery group, with statistically significant differences (*P* < 0.05). Ligamentum flavum hypertrophy predominantly involved:C4–C5 (81.3%), C3–C4 (62.5%), C5–C6 (56.2%). Preoperative T2-weighted MRI showed a mean thickness of 3.8 ± 0.6 mm (range: 3.1–5.2 mm).

**Table 1:** Comparison of basic parameters and perioperative information between the Two groups.

Index	Endoscopic group (*n* = 16)	Open group (*n* = 20)	Statistic (*t*/*χ*^2^)	*P* value
Gender (Male/Female)	10/6	11/9	0.206	0.650
Age (years)	57.7 ± 9.2	60.5 ± 10.2	0.859	0.397
BMI (kg/m^2^)	23.4 ± 1.7	24.1 ± 1.3	0.791	0.435
Duration of illness (months)	14.1 ± 3.7	13.9 ± 4.6	0.114	0.910
Smoking (Yes/No)	6/10	7/13	0.024	0.877
Hypertension (Yes/No)	7/9	11/9	0.450	0.502
Diabetes (Yes/No)	4/12	8/12	0.900	0.343
Follow-up time (months)	15.3 ± 1.9	13.9 ± 4.6	1.105	0.277
Surgical times (h)	1.6 ± 0.6	2.1 ± 0.2	3.333	0.002
Incision length (cm)	1.3 ± 0.1	9.5 ± 0.7	45.018	0.000
Blood loss (mL)	12.4 ± 7.4	64.3 ± 19.5	10.065	0.000
Hospital stay (days)	6.6 ± 1.1	8.6 ± 1.4	4.512	0.000

### 3.2 Clinical outcomes

Serial assessments of pain and functional outcomes are detailed in Table 2. Both groups showed significant improvements in VAS, JOA, and NDI scores at all postoperative time points (1, 3, 6, 12 months and final follow-up) compared with preoperative baselines (*P* < 0.05). Interestingly, the VAS score was better in the endoscopic group than in the open group at 1-month follow-up (VAS score 2.69 ± 0.79 vs. 4.40 ± 0.88 *P* < 0.05). There was no statistically significant difference in the rate of neurological improvement between the two groups at each follow-up time point.

**Table 2:** Comparison of VAS, JOA, JOA improvement rate, and NDI scores between the two groups preoperatively and at different postoperative time points.

Index	Endoscopic group (*n* = 16)	Open group (*n* = 20)	*t* value	*P* value
VAS score				
Preoperative	7.25 ± 0.86	7.05 ± 1.15	0.58	0.57
Postoperative 1 month	2.69 ± 0.79^a^	4.4 ± 0.88^a^	6.05	0.00
Postoperative 3 months	1.75 ± 0.45^a^	2.3 ± 0.80^a^	1.21	0.27
Postoperative 6 months	1.38 ± 0.50^a^	1.75 ± 0.64^a^	1.92	0.06
Postoperative 12 months	1.06 ± 0.68^a^	1.20 ± 0.52^a^	0.69	0.49
Final follow-up	0.56 ± 0.63^a^	0.80 ± 0.52^a^	1.24	0.22
*F* value	217.35	185.08		
*P* value	0.00	0.00		
JOA score				
Preoperative	6.69 ± 1.35	6.9 ± 1.55	0.43	0.67
Postoperative 1 month	13.31 ± 1.14^a^	13.15 ± 1.60^a^	0.34	0.73
Postoperative 3 months	15.00 ± 0.82^a^	14.80 ± 1.74^a^	0.42	0.67
Postoperative 6 months	15.19 ± 0.66^a^	15.45 ± 1.05^a^	0.87	0.39
Postoperative 12 months	15.56 ± 0.89^a^	15.95 ± 0.69^a^	1.47	0.15
Final follow-up	15.94 ± 0.68^a^	16.20 ± 0.77^a^	1.07	0.29
*F* value	216.07	147.03		
*P* value	0.00	0.00		
JOA improvement rate at final follow-up (%)	0.89 ± 0.06	0.91 ± 0.07	0.94	0.354
NDI (%)				
Preoperative	40.13 ± 5.24	38.7 ± 3.51	0.98	0.34
Postoperative 1 month	18.06 ± 3.71^a^	18.7 ± 4.55^a^	0.452	0.654
Postoperative 3 months	9.63 ± 2.36^a^	7.95 ± 3.27^a^	1.72	0.095
Postoperative 6 months	7.69 ± 1.85^a^	6.10 ± 1.48^a^	2.86	0.01
Postoperative 12 months	4.56 ± 1.86^a^	3.65 ± 1.46^a^	1.65	0.11
Final follow-up	2.56 ± 1.41^a^	2.55 ± 1.19^a^	0.03	0.97
*F* value	336.87	466.69		
*P* value	0.00	0.00		

a Indicates statistically significant difference compared with preoperative values (*P* < 0.05).

### 3.3 Changes in radiographic parameters

**Figure 2:**
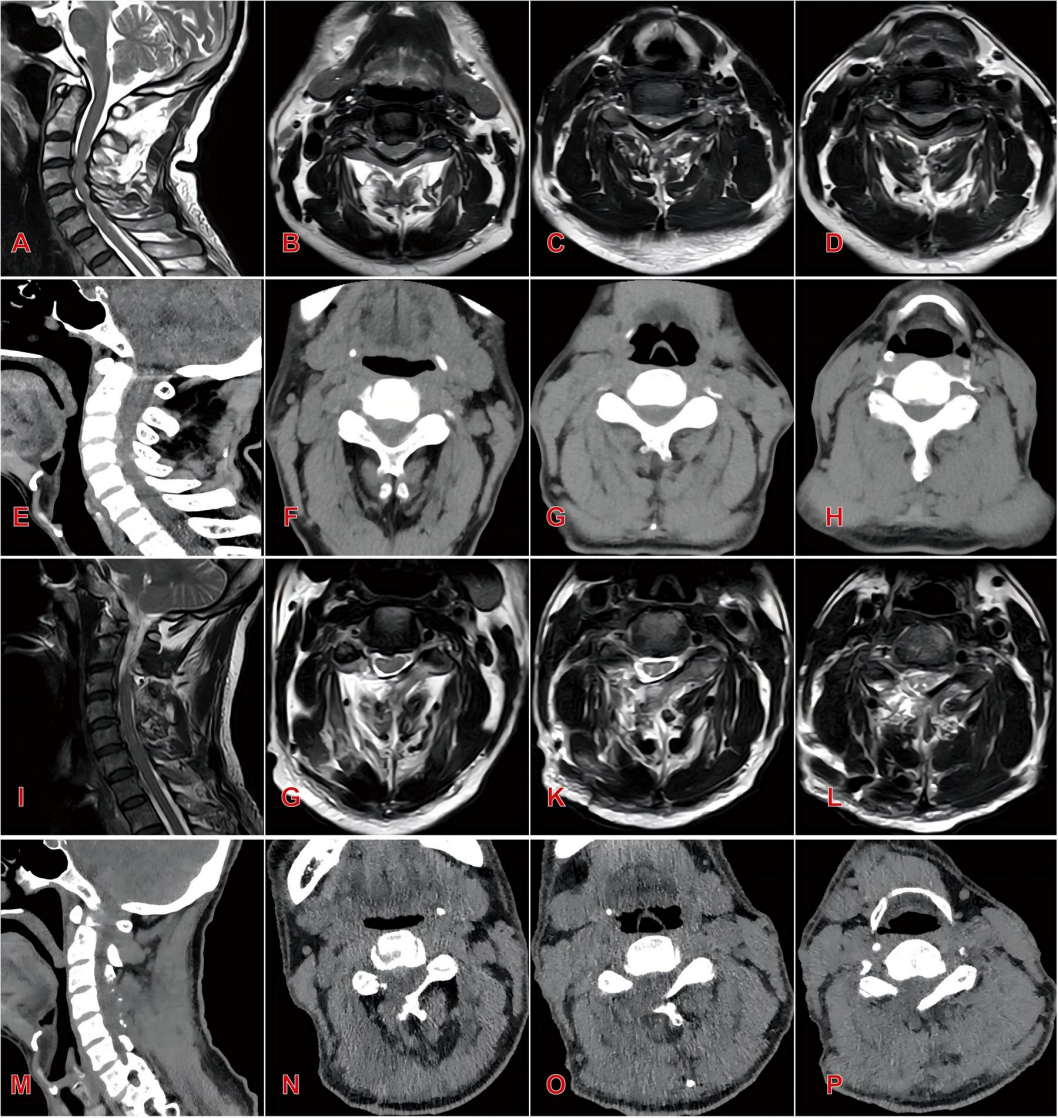
Typical case 1 of endoscopic group patients, a 55-year-old male with three-level cervical spinal stenosis (C3–C6) **(A)** preoperative sagittal MRI demonstrating multilevel cervical spinal canal stenosis; **(B–D)** preoperative axial MRI at C3–C4 **(B)**, C4–C5 **(C)**, and C5–C6 **(D)** levels; **(E)** preoperative sagittal CT reconstruction; **(F–H)** preoperative axial CT at C3–C4 **(F)**, C4–C5 **(G)**, and C5–C6 **(H)** levels; **(I)** postoperative sagittal MRI showing significant expansion of the spinal canal and restored cerebrospinal fluid patency; **(J–L)** postoperative axial MRI at C3–C4 **(J)**, C4–C5 **(K)**, and C5-C6 **(L)** levels; **(M)** postoperative sagittal CT reconstruction; **(N–P)** postoperative axial CT at C3–C4 **(N)**, C4–C5 **(O)**, and C5–C6 **(P)** levels.

Radiographic parameters of both groups preoperatively, postoperatively, and at final follow-up are summarized in Tables 3, 4. Both groups showed significant improvement in spinal canal area at the affected levels postoperatively (*P* < 0.05). However, the open group exhibited significantly higher segmental Pavlov ratios than the endoscopic group at both postoperative and final follow-up assessments (*P* < 0.05). No statistically significant intergroup differences were detected in C2–C7 Cobb angles pre- or postoperatively (*P* > 0.05). Notably, at final follow-up, the Endoscopic Group demonstrated significantly smaller C2–C7 Cobb angles compared to the Open Group (13.57 ± 2.29° vs. 16.34 ± 2.95°, *P* < 0.05). Similarly, T1 slope angles showed no significant intergroup differences pre- or postoperatively (*P* > 0.05). However, at final follow-up, the endoscopic group maintained significantly reduced T1 slopes relative to the open group (22.62 ± 1.51° vs. 25.24 ± 2.41°, *P* < 0.05). Representative case 1 images are presented in Figure 2.

**Table 3:** Comparison of radiographic parameters between the two groups.

Index	Endoscopic group (*n* = 16)	Open group (*n* = 20)	*t* value	*P* value
Cervical C2–C7 Cobb angle (°)				
Preoperative	16.78 ± 3.32	16.64 ± 3.07	0.14	0.89
Postoperative	16.71 ± 3.43	16.16 ± 2.9	0.52	0.61
Final follow-up	13.57 ± 2.29^a^	16.34 ± 2.95	3.08	0.01
*F* value	5.73	0.13		
*P* value	0.006	0.87		
T1 slope (°)				
Preoperative	23.37 ± 1.95	23.11 ± 1.98	0.79	0.69
Postoperative	23.25 ± 1.90	24.02 ± 2.3	1.08	0.28
Final follow-up	22.62 ± 1.51	25.24 ± 2.41^a^	5.95	0.00
*F* value	0.79	4.56		
*P* value	0.45	0.01		

a *P* < 0.05, compared with pre-operation.

**Table 4:** Comparison of pavlov ratios at each segment between the two groups.

Index	Time point	Endoscopic group (*n* = 16)	Open group (*n* = 20)	*t* value	*P* value
C3 Pavlov ratio	Preoperative	0.62 ± 0.08	0.69 ± 0.08	1.83	0.08
	Postoperative	0.85 ± 0.10^a^	0.95 ± 0.06^a^	2.97	0.007
	Final follow-up	0.84 ± 0.05^a^	0.93 ± 0.07^a^	3.27	0.003
	*F* value	14.91	89.61		
	*P* value	0.00	0.00		
C4 Pavlov ratio	Preoperative	0.63 ± 0.08	0.60 ± 0.06	1.47	0.15
	Postoperative	0.84 ± 0.06^a^	1.01 ± 0.07^a^	6.58	0.00
	Final follow-up	0.83 ± 0.06^a^	1.00 ± 0.06^a^	7.64	0.00
	*F* value	38.09	280.27		
	*P* value	0.00	0.00		
C5 Pavlov ratio	Preoperative	0.65 ± 0.08	0.68 ± 0.11	1.09	0.28
	Postoperative	0.85 ± 0.06^a^	1.01 ± 0.09^a^	6.50	0.00
	Final follow-up	0.86 ± 0.05^a^	0.99 ± 0.09^a^	4.99	0.00
	*F* value	62.27	69.84		
	*P* value	0.00	0.00		
C6 Pavlov ratio	Preoperative	0.65 ± 0.07	0.66 ± 0.11	0.22	0.83
	Postoperative	0.88 ± 0.04^a^	0.99 ± 0.09^a^	4.13	0.00
	Final follow-up	0.86 ± 0.05^a^	0.98 ± 0.08^a^	4.49	0.00
	*F* value	61.05	76.52		
	*P* value	0.00	0.00		

a *P* < 0.05, compared with pre-operation.

### 3.4 Complications

Two patients in the open group developed postoperative unilateral deltoid and biceps weakness with pain in the C5 dermatome, consistent with C5 nerve root palsy. One additional patient in this group experienced axial pain. All three received pharmacological management with corticosteroids, dehydrating agents, and anti-inflammatory analgesics to alleviate nerve root edema and irritation. Postoperative management included drainage system placement combined with therapeutic interventions: intravenous hydration, neurotrophic pharmacotherapy, and bed rest. In the Endoscopic Group, one patient exhibited transient bilateral upper limb weakness, which resolved with dehydrating agents and neurotrophic drugs by discharge. The authors considered that factors such as water pressure impact during surgery and prolonged surgery time may have caused transient nerve damage. At the 6-month follow-up, complete neurological recovery was confirmed. No revision surgery was required in either cohort.

## 4 Discussion

The open-door laminoplasty was first pioneered by Hirabayashi and has been continuously improved as an effective surgical procedure for treating multilevel spinal canal stenosis (7, 15). However, this approach carries inherent limitations that rongeur removal of the outer cortical bone at the hinged-side compromises structural integrity, elevating fracture risk (16). Subsequent segmental instability may manifest as postoperative neck pain or spinal cord dysfunction in patients. As spinal endoscopy theory and equipment have continuously improved, the range of applications is growing. Previous literature reported a 56-year-old male patient with C2–3 cervical spinal canal stenosis caused by calcification of the ligamentum flavum, who underwent large-channel spinal endoscope laminotomy decompression and recovered well after the operation (17). Similarly, D. Carr et al. demonstrated a 90.7% overall excellent rate following endoscopic unilateral laminotomy for bilateral decompression in 10 severe cervical stenosis cases (18). These studies indicate that large-channel spinal endoscope laminotomy decompression techniques is feasible in the treatment of single-level cervical spinal stenosis, effectively alleviating symptoms of cervical spinal cord compression, and laying the foundation for its application in multi-levels cervical spinal stenosis.

This study compared the efficacy and differences between large-channel spinal endoscope cervical laminotomy decompression and open-door laminoplasty for multilevel cervical stenosis cases. The endoscopic approach minimizes iatrogenic injury through enhanced intraoperative visualization and precision, preserving posterior muscular attachments and facet joint integrity which is particularly critical given that more than 50% of intervertebral joint injuries lead to a significant decrease in cervical spine stability. Notably, while the study's follow-up period (average: 13.6–15.3 months) was relatively brief and insufficient for long-term postoperative monitoring, the observed radiological trends still provided valuable predictive insights. Radiographic studies revealed a smaller C2–C7 Cobb angle in the endoscopic group (13.57 ± 2.29 vs. 16.34 ± 2.95 in the open group, *P* < 0.05) and a decreased T1 slope at final follow-up compared to preoperative baseline values, whereas the open group exhibited an increased T1 slope (*P* < 0.05). This divergence reflects open group's substantially greater trauma to posterior musculoligamentous complexes and joints, predisposing to postoperative kyphosis deformity, which is consistent with the research conducted by Fu et al. (19).

Importantly, although the spinal canal area in the endoscopic group was smaller than that in the open group after surgery and the Pavlov ratio at each segment was also lower than that in the open group, it is interesting to note that both groups demonstrated significant improvements in VAS, JOA, and NDI scores at all post-operative time points compared with their pre-operative status, with no significant difference observed in the JOA improvement rate between the two groups after surgery. This indicates that both surgical approaches are effective in relieving symptoms and promoting functional recovery. However, when considering other factors such as hospital stay duration and hospitalization costs, the endoscopic group patients exhibit superiority over the open group in terms of patients’ overall post-operative satisfaction.

Open-door laminoplasty for multilevel cervical spinal canal stenosis requires resection of ≥3 segments of the laminae to utilize the bowstring biomechanical effect for spinal cord decompression (15, 20). However, this extensive approach inherently risks iatrogenic structural compromise, correlating with higher complication rates. In this study, two patients in the open group developed C5 nerve root palsy and one patient developed axial pain symptoms after surgery. Following Open-door laminoplasty, the spinal cord was adequately decompressed, and the dura mater drifted backward, which results in traction on the nerve roots. C5 nerve root paralysis occurred because the C5 nerve root is relatively short (21, 22). Pharmacological management (corticosteroids + dehydrating agents) effectively reduced root edema. Studies have found that more than 70% of patients with C5 nerve root palsy can fully recover muscle strength within 4–5 months after surgery (23). Large-channel spinal endoscope cervical laminotomy decompression can precisely remove compressive tissue and expand the spinal canal area through visual guidance. At the same time, it reduces intraoperative bleeding and injury. It has the advantages of minimal injury, less bleeding, quick recovery, and high patient satisfaction.

Precise decompression of spinal endoscopy is achieved by resecting the caudal margin of the superior lamina and rostral margin of the inferior lamina for the treatment of single-level cervical spinal stenosis. For multilevel cervical spinal stenosis, it is usually necessary to completely remove the unilateral lamina of the middle segments. This not only helps to fully expand the spinal canal area, but also reduces the risk of residual bone fractures and inadvertent spinal cord injury.

In addition, the authors have the following insights regarding the details of the surgical procedure: (1) The size of the incision is critical to the success of the surgery. The authors typically select a 1 cm incision. An incision that is too large may affect the tightness of the large channel. When inserting the working channel, it should be rotated clockwise to avoid displacement or instability. (2) When removing the lamina, the authors make initial lamina thinning with horizontal burring force. Then use the laminar rongeur to gently remove the inner layer of cortical bone, gradually exposing the ligamentum flavum. This is very important and can effectively prevent diamond abrasor damage to the spinal cord. It should be emphasized that spinal endoscopic surgery entails a steep learning curve and demands advanced surgical skills for the operator. Notably, all cases included in this study were performed after the team completed their learning curve, ensuring that all endoscopic group patients followed standardized surgical techniques and protocols. In addition, the indications for this surgical operation are relatively limited. The surgical indications should be strictly controlled to avoid poor postoperative efficacy. This study has several limitations: First, the small sample size and short follow-up duration constrain statistical power. Second, the assessment of cervical stability mainly relies on two-dimensional measurement methods. Future investigations should employ three-dimensional reconstruction to quantify dynamic changes in spinal canal volume and biomechanical parameters. Furthermore, as a single-center retrospective study, the generalizability of findings may be limited. Prospective randomized controlled trials are warranted to corroborate these conclusions.

## 5 Conclusions

In summary, for the treatment of multilevel cervical spinal stenosis, large-channel spinal endoscope unilateral laminotomy decompression demonstrates comparable short-term efficacy to open-door laminoplasty. Furthermore, the endoscopic approach offers distinct advantages including minimally invasive access, reduced intraoperative blood loss and accelerated postoperative recovery.

## Data Availability

The original contributions presented in the study are included in the article/Supplementary Material, further inquiries can be directed to the corresponding authors.
